# Commentary: *Theileria* Parasites Secrete a Prolyl Isomerase to Maintain Host Leukocyte Transformation

**DOI:** 10.3389/fmed.2018.00120

**Published:** 2018-04-27

**Authors:** Ruben Fernandes, Stephanie Ferreira, Mónica C. Botelho

**Affiliations:** ^1^I3S, Instituto de Investigação e Inovação da Universidade do Porto, Porto, Portugal; ^2^Escola Superior de Saúde, Instituto Politécnico do Porto, Porto, Portugal; ^3^Facultad de Medicina, Universidad Santiago de Compostela, Santiago de Compostela, Spain; ^4^Department of Health Promotion and Chronic Diseases, INSA, National Institute of Health Dr. Ricardo Jorge, Porto, Portugal

**Keywords:** *Theileria*, parasites, cancer, miR-155, *Schistosoma haematobium*

The genus *Theileria* comprises apicomplexan parasites that are tick-transmitted and infect ruminants with important trade and industry impact in endemic countries (1). Infectious agents build up elaborate machinery to work together by means of host cell pathways and takeover their genetic and epigenetic mechanisms to modify phenotypic status of host cells. Among the Apicomplexa phylum encompassing obligate intracellular parasites, the only genus that introduces its DNA into mammalian host cells is *Theileria*, which is known to cause veterinary and human diseases (2). Various species, including *Theileria parva* and *Theileria annulata*, infect leukocytes causing alterations in their phenotypes that are common with several cancers, remarkably immortalization, hyperproliferation, and spreading (1).

Pin1 is a distinctive peptidyl prolyl isomerize (PPIase) that catalyzes the cis/trans isomerization of peptidyl–prolyl peptide bonds of its substrate proteins by binding to their specific phosphorylated Ser/Thr-Pro (pSer/Thr-Pro) motifs. This modifies the conformation of target proteins and, therefore, has an effect on their stability, intracellular localization, and/or biological roles. The atypical overexpression of Pin1 is observed in a number of malignancies, which is linked with tumor cell proliferation, migration, and invasion (3). PIN1 is an enzyme with peptidyl-prolyl isomerase activity that catalyzes the *cis*/*trans* isomerization of peptide bonds flanked by proline and phosphorylated serine/threonine residues. By shifting the conformation of its protein substrates, PIN1 amplifies the behavior of vital proteins that support cell cycle progression and oncogenesis (4).

Marsolier et al. (2) became aware of *Theileria* Pin1 transcripts in B cells infected with *T. annulata* or *T. parva*. Confocal microscopy and immunoblot analysis placed the parasite Pin1 protein together in the host cell cytoplasm and nucleus (2). These authors affirm that the pooled results illustrate that *Theileria* produces a “bona fide” phosphorylation-dependent PPIase, which may well add to host cell transformation (2).

In the report of Marsolier et al. (2), the authors also studied c-Jun pathway. These authors showed that c-Jun is vital for *Theileria* transformation and host cell proliferation. In B lymphocytes infected in culture with *T. annulata* (TBL3 cells), c-Jun appears to be triggered by reduced ubiquitin ligase bovine protein FBW7 degradation more willingly than phosphorylation by JNK signaling. Later, establishment of a feedback loop, relating to c-Jun control of the miR-155 oncomiR, might generate an epigenetic exchange to sustain transformation and proliferation (2). This miR-155 may have biomarker value and can be easily detected by biosensors that our group already developed and detect its variation at attomolar levels (5).

In fact, host cell proliferation is an important hallmark of cancer (6). Our group has previously shown that cells treated with *Schistosoma haematobium* extracts increase proliferation (7).

It has also been demonstrated that schistosomes possess a PPIase activity (8). This protein might explain the increased proliferation observed in the cells treated with Schistosomes extracts (7). It will be as well appealing in the future to verify whether PIN1 from schistosomes also show evidence of a similar stimulating consequence of cell proliferation.

The other well-known cancer inducing parasite *Opisthorchis* and *Clonorchis* have also been shown to exhibit PPIase activity. *Clonorchis sinensis* adult in biliary ducts has been shown to release *C. sinensis* cyclophilin A, a protein with PPIase activity, into the liver and play a role in inflammation and biliary epithelium proliferation and adenomatoid hyperplasia (9).

Hutadilok et al. (10) have shown that a prolyl hydroxylase activity was observed in hamsters infected with the human liver fluke *Opisthorchis viverrini*. This activity accompanied an increase in liver collagen that is associated with an increase in the proliferation of fibroblasts (10).

In conclusion, infection with the eukaryotic protozoan *Theileria* parasite causes diseases of medical or economic importance like tropical theileriosis and East Coast Fever. This apicomplexan is an obligate intracellular parasite of host lymphocytes. Amidst this group of parasites, *Theileria* is the only genus known to transform their host cell. This parasite secretes a prolyl isomerase that promotes transformation of host cells through inhibiting c-JUN pathway. Given the striking similarities of how *Theileria* induces transformation of their host lymphocytes and tumorigenesis, these findings might have major implications for infection-induced cancer and the mechanisms that underlie this causality that might be common with other cancer-inducing parasites.

## Author Contributions

MB: planned, wrote, and revised. RF: wrote and revised. SF: revised.

## Conflict of Interest Statement

The authors declare that the research was conducted in the absence of any commercial or financial relationships that could be construed as a potential conflict of interest.

## References

[1] Tretina K Gotia HT Mann DJ Silva JC. *Theileria*-transformed bovine leukocytes have cancer hallmarks. Trends Parasitol (2015) 31:306–14. 10.1016/j.pt.2015.04.001 25951781

[2] Marsolier J Perichon M DeBarry JD Villoutreix BO Chluba J Lopez T *Theileria* parasites secrete a prolyl isomerase to maintain host leukocyte transformation. Nature (2015) 520:378–82. 10.1038/nature14044 25624101 PMC4401560

[3] Han HJ Choi BY Surh YJ Dual roles of Pin1 in cancer development and progression. Curr Pharm Des (2017) 23(29):4422–5. 10.2174/1381612823666170703164711 28671058

[4] Cheng CW Leong KW Ng YM Kwong YL Tse E. The peptidyl-prolyl isomerase PIN1 relieves cyclin-dependent kinase 2 (CDK2) inhibition by the CDK inhibitor p27. J Biol Chem (2017) 292:21431–41. 10.1074/jbc.M117.801373 29118189 PMC5766953

[5] Cardoso AR Moreira FTC Fernandes R Sales MGF. Novel and simple electrochemical biosensor monitoring attomolar levels of miRNA-155 in breast cancer. Biosens Bioelectron (2016) 80:621–30. 10.1016/j.bios.2016.02.035 26901459 PMC6366556

[6] Botelho MC Alves H Barros A Rinaldi G Brindley PJ Sousa M. The role of estrogens and estrogen receptor signaling pathways in cancer and infertility: the case of schistosomes. Trends Parasitol (2015) 31:246–50. 10.1016/j.pt.2015.03.005 25837311

[7] Botelho M Ferreira AC Oliveira MJ Domingues A Machado JC da Costa JM. *Schistosoma haematobium* total antigen induces increased proliferation, migration and invasion, and decreases apoptosis of normal epithelial cells. Int J Parasitol (2009) 39:1083–91. 10.1016/j.ijpara.2009.02.016 19285502

[8] Bugli F Khattab A Vigneti E Butler R Cioli D Klinkert MQ. Expression cloning and biochemical characterizations of recombinant cyclophilin proteins from *Schistosoma mansoni*. Protein Expr Purif (1998) 12:340–6. 10.1006/prep.1997.0852 9535701

[9] Wu W Chen J Zeng S Zhang Z Gan W Yu X Molecular cloning, expression, and characterization of cyclophilin A from *Clonorchis sinensis*. Parasitol Res (2011) 109:345–51. 10.1007/s00436-011-2262-2 21360097

[10] Hutadilok N Ruenwongsa P Upatham ES. Increased liver prolyl hydroxylase activity in hamsters infected with the human liver fluke *Opisthorchis viverrini*. Experientia (1983) 39:1004–5. 10.1007/BF01989773 6309553

